# Manic Episode or Dissociative Episode? The Importance of Differential Diagnosis in Maniform Symptomatology: A Case Report

**DOI:** 10.1192/j.eurpsy.2025.1809

**Published:** 2025-08-26

**Authors:** S. Castelao-Almodovar, A. Arce de la Riva, R. Albillos Perez, A. Pérez Balaguer, E. Gil Benito

**Affiliations:** 1 Centro de Salud Mental El Escorial, El Escorial; 2 H. U. Puerta de Hierro, Majadahonda, Spain

## Abstract

**Introduction:**

Dissociative episodes are characterized by disruptions in consciousness, identity, or perception of reality and can be confused with other psychiatric disorders, such as manic episodes. This case describes a patient with both manic-like and dissociative symptoms, highlighting the need for a precise differential diagnosis. The case emphasizes how prolonged stress and psychosocial factors triggered a dissociative crisis, which improved significantly with anxiolytic and antidepressant treatment, a key factor in clarifying the diagnosis.

**Objectives:**

To describe a case of dissociative episode with manic-like symptoms, emphasizing the importance of differential diagnosis and appropriate treatment.

**Methods:**

We present the case of a 43-year-old woman who was admitted to the emergency department following an acute dissociative episode. The patient arrived with a behavioral disturbance that had begun abruptly a few days earlier. She reported experiencing feelings of clairvoyance, mystical thoughts, and the sensation of receiving “alphanumeric codes,” interpreting gestures and everyday objects as messages with special meaning. During this episode, she exhibited behavioral disorganization, prolonged insomnia, euphoria, and a sense of hyperactivity, with impulses to compulsively reorganize her surroundings. These symptoms escalated until the patient, recognizing she could not control the situation, alerted her husband, who decided to take her to the emergency department due to the severity of her condition.

**Results:**

Disorganized behavior, with accelerated and delusional speech and megalomaniacal thoughts were present. Complementary tests were performed, ruling out organic causes. After administering midazolam for imaging procedures and initiating a fixed regimen of Lorazepam, the symptoms improved markedly, allowing for a more accurate assessment. The episode was interpreted as dissociative, with identified triggers including work-related stress, her relationship with her daughter, and the recent death of a friend. Pharmacological treatment with anxiolytics and antidepressants proved effective, leading to significant improvement in the following weeks, with resolution of delusions and insomnia.

**Conclusions:**

This case highlights the importance of differential diagnosis between a dissociative episode and a manic episode, given the shared symptoms such as megalomaniacal delusions, insomnia, and euphoria. The rapid response to anxiolytic and antidepressant treatment suggests that the episode was more related to a stress-induced dissociative disorder rather than a bipolar affective disorder that would have required mood stabilizer treatment. This therapeutic approach resulted in a complete remission of symptoms, demonstrating the importance of a treatment plan tailored to the patient’s specific needs.

**Disclosure of Interest:**

None Declared

